# Chemotherapy curable malignancies and cancer stem cells: a biological review and hypothesis

**DOI:** 10.1186/s12885-016-2956-z

**Published:** 2016-11-21

**Authors:** Philip Savage

**Affiliations:** BCCA, Vancouver Island, Victoria, BC Canada

**Keywords:** Cancer stem cells, Chemotherapy, Resistance, Hierarchy, Stochastic, Apoptosis

## Abstract

**Background:**

Cytotoxic chemotherapy brings routine cures to only a small select group of metastatic malignancies comprising gestational trophoblast tumours, germ cell tumours, acute leukemia, Hodgkin’s disease, high grade lymphomas and some of the rare childhood malignancies.

We have previously postulated that the extreme sensitivity to chemotherapy for these malignancies is linked to the on-going high levels of apoptotic sensitivity that is naturally linked with the unique genetic events of nuclear fusion, meiosis, VDJ recombination, somatic hypermutation, and gastrulation that have occurred within the cells of origin of these malignancies.

In this review we will examine the cancer stem cell/cancer cell relationship of each of the chemotherapy curable malignancies and how this relationship impacts on the resultant biology and pro-apoptotic sensitivity of the varying cancer cell types.

**Discussion:**

In contrast to the common epithelial cancers, in each of the chemotherapy curable malignancies there are no conventional hierarchical cancer stem cells. However cells with cancer stem like qualities can arise stochastically from within the general tumour cell population. These stochastic stem cells acquire a degree of resistance to DNA damaging agents but also retain much of the key characteristics of the cancer cells from which they develop. We would argue that the balance between the acquired resistance of the stochastic cancer stem cell and the inherent chemotherapy sensitivity of parent tumour cell determines the overall chemotherapy curability of each diagnosis.

**Summary:**

The cancer stem cells in the chemotherapy curable malignancies appear to have two key biological differences from those of the more common chemotherapy incurable malignancies. The first difference is that the conventional hierarchical pattern of cancer stem cells is absent in each of the chemotherapy curable malignancies.

The other key difference, we suggest, is that the stochastic stem cells in the chemotherapy curable malignancies take on a significant aspect of the biological characteristics of their parent cancer cells. This action includes for the chemotherapy curable malignancies the heightened pro-apoptotic sensitivity linked to their respective associated unique genetic events.

For the chemotherapy curable malignancies the combination of the relationship of their cancer stem cells combined with the extreme inherent sensitivity to induction of apoptosis from DNA damaging agents plays a key role in determining their overall curability with chemotherapy.

## Background

Despite the introduction of a significant number of new cancer therapeutics that target specific molecular pathways within malignant cells, the use of DNA damaging cytotoxic chemotherapy currently remains the mainstay in the management of most malignancies [1].

In the majority of metastatic malignancies, DNA damaging cytotoxic chemotherapy can reduce the disease bulk, improve symptoms and extend life [2]. However, despite these often significant benefits from treatment, curative treatment with chemotherapy is not a realistic outcome for patients with the common metastatic malignancies. In contrast in a select group of relatively rare malignancies, curative treatment with chemotherapy drugs is the expectation even for the patients with widely disseminated and high tumor burden disease [3].

Beginning in the 1950s and fully established by the 1980s there has been the development of routine curative chemotherapy treatment for most patients with acute leukemia, high grade non-Hodgkin’s lymphoma (NHL), Hodgkin’s disease, testicular and ovarian germ cell tumors, the gestational trophoblast tumors and for many cases of the rare childhood malignancies [2, 4]. In current practice the cure rates for a number of these diagnoses is in excess of 90%, with the first line cytotoxic drug treatment of each malignancy comprised entirely of drugs developed by the early 1980s. Whilst there is significant short term toxicity from chemotherapy, generally treatment is relatively well tolerated and patients are routinely cured. After treatment patients return to normal health and appear to have no significant long term toxicity to any cell types or to their tissue specific healthy somatic stem cells [5].

There is clearly a significant clinical and biological divide between these rarer malignancies that can be routinely cured with cytotoxic chemotherapy and the majority of the more common cancers that are incurable in the metastatic setting and only relatively rarely cured even in the adjuvant setting. The biological explanation for this divergence presents a significant challenge to both understating of the cellular processes involved and to the development of more effective approaches to care [6, 7].

With such a routine but dramatic and reproducible divide in chemotherapy sensitivity and treatment outcomes between these differing tumor cell types, the conventional explanations that ascribe chemotherapy resistance to the two main continuous variable parameters of the rate of tumour cell growth and the development of genetic mutations that lead to resistance are perhaps worthy of an updated review.

Historically the concept of the inherent chemotherapy sensitivity of tumour cells and the concept of ‘log kill’ originated in early models that used murine leukemia as the model [8, 9]. As we will review later, acute leukemia may well be deeply unrepresentative of the biology of the majority of tumor cell types. As a result the interpretation of experimental data from this cell type should be handled with caution with regard to the unique biological properties of these early B cell malignancies compared to that of the more common epithelial malignancies.

We have recently suggested an alternate biological explanation for the extreme chemotherapy sensitivity of the chemotherapy curable malignancies. The theory centres on the close association between the cells of the chemotherapy curable malignancies and the occurrence of their unique genetic events of nuclear fusion, meiosis, variable-diversity-joining (VDJ) recombination, and somatic hypermutation (SHM) each of which has naturally upregulated apoptotic pathways as shown in Table 1 [3, 4]. In this updated review we will examine the relationship between cancer stem cell biology, the developmental pathways of the chemotherapy curable malignancies and the potential impact these have on the natural apoptotic sensitivity of the cancer stem cells in the differing diagnoses.

**Table 1:** Cancer stem cell structure, associated unique genetic events and chemotherapy curability

Metastatic malignancy	Cancer stem cell model	Associated unique genetic event	Chemotherapy curability
Common epithelial cancers	Hierarchical	None	-
Gestational trophoblast tumours	Non-hierarchical	Nuclear fusion	+++
Germ cell tumours	Non-hierarchical	Meiosis	+++
Childhood malignancies	Non-hierarchical	Gastrulation	+
B and T cell malignancies			
Acute undifferentiated leukemia	Hierarchical	None	-
Acute lymphocytic leukemia	Non-hierarchical	VDJ	+++
ALK + ve T Cell lymphoma	Non-hierarchical	VDJ	++
Mantle cell lymphoma	Non-hierarchical	None	-
Diffuse large B Cell lymphoma	Non-hierarchical	SHM	++
Burkitt’s lymphoma	Non-hierarchical	SHM	++
Hodgkin lymphoma	Non-hierarchical	SHM	++
CLL	Non-hierarchical	None	-
Myeloma	Non-hierarchical	None	-
Mature T cell malignancies	Non-hierarchical	None	-

The relationship of the cancer stem cell model, of being either hierarchical as in the common epithelial cancers or non-hierarchical without somatic stem derived cancer stem cells, the associated unique genetic events and the associated degree of chemotherapy curability are shown. For simplicity chemotherapy curability is shown on a zero to 3 star range

### Cancer stem cells; Hierarchical and stochastic

The existence and important role of cancer stem cells appears to support many of the clinical observations on malignant cells and their responses to treatment. Whilst their existence was originally suggested in the 19^th^ century [10], cancer stem cells were first identified in the 1990s, in acute myeloid leukemia (AML) [11, 12]. Further studies have now observed cancer stem cells in a wide range of malignancies including glioblastoma, breast, endometrial, pancreatic, prostate, lung, colon cancers and a range of other tumor types [13–17]. Overall cancer stem cells are characterised as having distinct key properties including: self renewal, the ability to differentiate, active anti-apoptotic pathways, the expression of CD44, aldehyde dehydrogenase, CD133 and other markers also expressed by normal tissue specific somatic stem cells [18].

The importance of tissue specific somatic stem cells on cancer biology and therapeutics is twofold. Firstly, the existence of tissue specific stem cells allows for cells to be sufficiently long lived to be able to accumulate the number of mutations required to develop the malignant phenotype [18]. The second characteristic of tissue specific stem cells is that they have an intrinsically biologically damage resistant phenotype that allows the tissue specific stem cells to be resistant to natural adverse challenges. This phenotype is that result of two processes, the first the presence of multiple systems including ATP-binding cassette (ABC) transporter proteins, that help protect the stem cell from any genotoxic agents [19, 20] and secondly the low level of apoptotic response to any DNA damage results in the resistance to chemotherapy treatment that may kill cancer cells and more mature normal healthy cells.

The resistance to chemotherapy of the normal tissue specific somatic stem cells appears to be routinely shared by the stem cells that have undergone malignant transformation and have become cancer stem cells [21]. It is believed that the natural resistance of the cancer stem cells to chemotherapy treatment may contribute to the underlying chemotherapy resistance of the common malignancies [14, 22, 23].

The main cancer stem cell model relates to cancer stem cells that form a hierarchy in giving rise to the tumour cells, following the linear progression from somatic stem cell to cancer stem cell and then to cancer cell. However there is also evidence that cells with some similar properties of stemness can arise from within the tumour cell pool in the stochastic cancer stem cell model. However these cancer stem cells having a different aetiology to hierarchical stem cells and the potential to have different biological characteristics. These two approaches to cancer stem cells are not mutually exclusive with stochastic cancer stem cells being reported in colon cancer that is also supported by a hierarchical cancer stem system [24].

### The malignant phenotype and inhibition of developmental progression

By definition the development of the malignant phenotype dramatically affects the biology of the cell with a major impact on the inhibition of normal patterns of cellular differentiation, senescence, ageing and programmed cell death [25]. The result of these processes is to lead to the accumulation of excess cells, a lack of control of numbers, abnormal development patterns and the inhibition of apoptosis that is normal part of the natural history of healthy cells.

The most dramatic impact of the onset of the malignant phenotype on altering cellular development can be seen in B and T cell malignancies which arise at a number of differing points along the normal but complex linear lymphocyte development pathways. In these malignancies, the malignant cells appear to become developmentally frozen with no further progression in the normal inear development of these cells from the time of the onset of the malignant phenotype [26]. As a result B cell malignancies that arise at differing points in B cell development can give rise to a wide spectrum of malignancies ranging from acute lymphocyctic leukemia (ALL), through chronic lymphocytic leukemia (CLL), mantle cell lymphoma, diffuse large B cell lymphoma (DLBCL), Hodgkin’s disease, follicular lymphoma, and on to multiple myeloma. The actual diagnosis and treatment related prognosis being dependant on the point in B cell development when the malignant phenotype is established and further cellular development halted [27].

More recently we have postulated how the impact of the onset of malignancy on freezing the cells normal developmental progression may leave malignant cells developing at certain normally transient but key developmental points with fixed heightened physiological pro-apoptotic potential [3]. For each of the chemotherapy curable malignancies there appears to be a close association with a unique physiological genetic event and their naturally associated relevant pro-apoptotic pathways. These events are nuclear fusion for gestational trophoblast tumors, meiosis for germ cell tumours, VDJ recombination for ALL and anaplastic lymphoma kinase (ALK) + ve T cell NHL, somatic hypermutation (SHM) for DLBCL, Hodgkin’s disease and Burkitt’s lymphoma and gastrulation for the rare childhood malignancies. Based on the assumption that the onset of malignancy occurs closely related to these events, we have postulated the persistence of the naturally upregulated pro-apoptotic machinery associated with these events remaining active and that this may be a likely major contributor to the ease of cure of these malignancies with chemotherapy treatment [4].

### Cancer cell of origin, ABC transporter proteins and chemotherapy sensitivity

Alongside the major contribution made to chemotherapy responses of the chemotherapy curable malignancies by the apoptotic sensitivity associated with their associated unique genetic developments, the ABC transport proteins also plays an important role in determining chemotherapy responses [28, 29].

A number of studies have indicated that the expression of this family of molecules is associated with a reduction in the efficacy of chemotherapy as a result of the increased efflux of drugs from cancer cells [30, 31]. However despite this important role, clinical developments to reverse this impact have not to date been of significant success [32–35].

The relationship between stem cells, the cancer cell of origin, the expression of the ABC system and the response to chemotherapy treatment in the chemotherapy curable malignancies helps to define the unique biology of these cells.

The ABC protein system is widely expressed in conventional tissue specific stem cells, where it plays a key role in protecting these cells from genotoxic stress [36]. Similarly in epithelial cancer stem cells, which are derived from tissue specific stem cells, ABC proteins are documented to be persistently expressed [37–39]. In the majority of solid tumours further increases in ABC protein expression levels have been associated with declining sensitivity to chemotherapy [40, 41].

However, as previously discussed, in the chemotherapy curable malignancies, there are no standard hierarchical cancer stem cells derived from the tissue specific stem cells and each of these malignancies arises from a transient developmental cell. It is apparent that these transient developmental cells have very differing patterns of ABC protein expression compared to tissue specific stem cells and this status appears to impact significantly on the expression of ABC proteins in malignancies derived from them.

In normal trophoblast cells, assessment of the expression and functional activity of ABC transporter genes in murine embryos indicate that activity of these efflux systems is not present immediately post nuclear fusion but only becomes active by day 6 [42]. Pathological studies demonstrate that in molar pregnancies there is only focal and much lower levels of the multi-drug resistance transporter ABCB5 expression than is seem in normal first trimester placentas [43]. This observation suggests that the failure of molar pregnancies to successfully undergo nuclear fusion and subsequent cellular differentiation is associated with an inhibition of the cellular development that would normally include the normal increase in expression of ABC proteins.

There is less data on the expression of the ABC family proteins in choriocarcinoma and placental site trophoblast tumours, however the limited data indicates that the majority of cases of choriocarcinoma or PSTT do not express ABCB5 [43].

B and T cell malignancies arise from differing transient cell types occurring along the lymphocyte development pathways [26]. The level of ABC protein expression at these points can vary significantly and this can impact on the lasting phenotype and chemotherapy sensitivity of the resultant malignancies. In normal B cells, studies have indicated high levels of functional activity for the efflux pump systems in HSCs but that these levels decline sharply in cells as they become committed as 34+/38+, 34+/33+, or 34+/10+ progenitor cells [44]. A wider review of ABC protein expression indicates that their activity varies across B cell development from a peak as HSC then falling during B cell development and then rising again in plasma cells [45].

Data suggests that this variation in ABC protein activity during normal B cell development is reflected in the various malignancies arising along the course of lymphocyte development. A number of studies have indicated that there the expression of ABC family members is significantly lower in both B cell and T cell ALL than in HSCs [46]. This reduction in the ABC activity marks that the ALL cells have, on entering VDJ rearrangement, moved away from the phenotype of the chemotherapy resistant HSC and the low level of ABC protein expression may in part be linked to the sensitivity of ALL to chemotherapy.

In Hodgkin’s lymphoma which is believed to arise from germinal centre B cells, the majority of cases do not express ABC proteins. Of note a more recent study has indicated that the approximately 30% of Hodgkin’s cases that do express the ABCC1 protein have a tendency to a worse prognosis [47]. Similarly in DLBCL expression of the ABC system is highly variable between cases and the outcome is worse in patients with higher levels of expression [48].

In contrast in CLL, levels of ABC proteins are higher than in DLBCL [49] and myeloma which develops from cells in the final stage of B cell development expresses ABC proteins at higher levels which can rise further on exposure to chemotherapy [50].

In testicular cancer characteristically the majority of ABC proteins are expressed at only very low levels, with significant levels of expression only seen in tumours that have demonstrated chemotherapy resistance [51]. The presumed cell of origin of testicular cancer is intraepithelial germ cell neoplasia which arises from a defect in gonocyte development. There is limited information on the expression of ABC proteins in these cells, but these cancer precursor cells are, unlike hierarchical cancer stem cells, very sensitive to chemotherapy [52] and it is likely that low or absent ABC expression will occur in these unique cells.

Reviewing the chemotherapy curable malignancies as a group it is apparent that they have this additional shared characteristic of generally low levels of ABC protein expression. This finding is intrinsically linked to particular cells of origin of these malignancies being transient developmental cells. Each of these developmental cells themselves have low ABC expression, rather than the high levels that are seen in hierarchical cancer stem cells. The relative role that these low levels of ABC expression plays compared to the impact of the unregulated apoptotic pathways associated with the unique genetic events occurring in these cells is an area to debate and hopefully one with interesting data to come.

## Discussion

### Cancer stem cells and chemotherapy curability

**Fig. 1:**
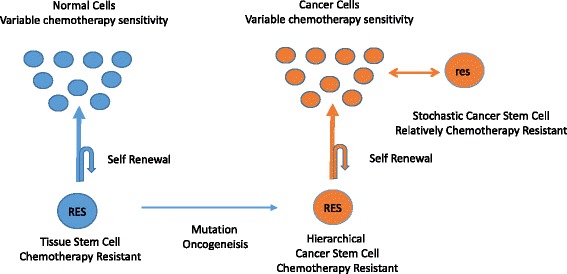
The Hierarchical Cancer Stem Model. In this simplified model, the standard somatic stem cells, give rise to healthy cells and also maintain their stem cell pool. In the epithelial cancers the somatic stem cells can give rise to the hierarchical cancer stem cells that retains much of the properties of the somatic stem cell, but gives rise to cancer cells. In some malignancies, cells with stem-like qualities can arise from within the tumour cell pool in a stochastic model

In the common epithelial malignancies the conventional structure of the cancer stem cell is the hierarchical system as shown in Fig. 1. In this system the cancer stem cells arise from somatic stem cells and give rise to their progeny of malignant cells which go on to form the bulk of the tumour. In response to chemotherapy treatment the progeny cancer cells in some diagnoses can respond dramatically to treatment, as seen in small cell lung cancer or epithelial ovarian cancer. However the cancer stem cells do not share the sensitivity to chemotherapy of their progeny tumour cells, but rather have the characteristic chemotherapy resistance of the tissue specific somatic stem cells. As a result the cancer stem cells survive the chemotherapy treatment and can then serve to repopulate the tumour and the cancer is hence destined to clinically relapse [21].

In contrast in the chemotherapy curable malignancies the role and relationship of potential cancer stem cells appears to be significantly different in both the hierarchy of the cancer stem cell/cancer cell relationship and also of crucial importance in the characteristics of the cancer stem cell with regards to sensitivity to chemotherapy. Cancer stem cells in the chemotherapy curable malignancies have proven to be challenging to study but cells with stem like properties have been reported in most of these diagnoses. As discussed below it appears that in each case of the chemotherapy curable malignancies they do not have standard hierarchical cancer stem cell structure, however they do have a population of cells that have developed cancer stem cell qualities when arising from the malignant cells themselves in a stochastic or non-hierarchical model.

The origin of the cancer stem cells in these malignancies may have a dramatic impact on their characteristics. Whilst the hierarchical stem cells will share many characteristics with the tissue specific somatic stem cells from which they arise, the stochastic stem cells arising from within the cancer cell pool whilst taking on some of the acquired characteristics of stemness, will also take on some of the primary biological properties of the malignant cell [53]. We would argue that the stochastic cancer stem cell in taking on key biological traits of the parent cancer cell from which they arise would include assuming the inherent apoptotic sensitivity of the malignant cell linked to the unique genetic event that is associated with each chemotherapy curable cancer type [4].

### In acute lymphocytic leukemia

ALL characteristically arises in pro-B cells that have or are undergoing VDJ recombination of their immunoglobulin genes [26]. B cells at this point in development have been shown to be dramatically more sensitive, compared to myeloid cells and mature B cells, to DNA damage from radiation or chemotherapy [54].

Research into the existence and potential characteristics of cancer stem cells in acute lymphocytic leukemia (ALL) has been an area of considerable endeavour. A number of studies have indicated that there is no cancer stem cell hierarchy in ALL [28, 55, 56] but that the majority of ALL cells have the potential stem cell properties of being able to self-renew and to transplant the disease in xenograft models [57]. As in the other B cell and T cell malignancies, the events of VDJ recombination breaks the link from the original chemotherapy resistant tissue HSC. As the stochastic cancer stem cells in ALL arise from the ALL cells themselves, we would postulate they will have the VDJ associated apoptotic sensitivity that is present in pro-B cells still active. This will give the ALL cells and their stochastic stem cell their great sensitivity to chemotherapy and results in the ability to have routine curative chemotherapy treatment as both cancer cells and the ALL stochastic cancer stem cells will remain exquisitely sensitive to DNA damage induced apoptosis.

### In non-Hodgkin’s lymphoma and Hodgkin’s disease

Lymphoma presents a range of diagnoses of B cell malignancies arising from mature B cells that have completed VDJ recombination. Cells with stem like properties have been identified in a number of types of mature B cell malignancies including chronic lymphocytic leukemia (CLL), follicular lymphoma and myeloma [58–60]. In keeping with the earlier observations, these stem cells all appear to arise from within the pool of the relevant malignant cells and do not have the conventional hierarchical stem cell structure that is seen in epithelial malignancies [58]. Similarly in Hodgkin’s disease, B cells able to generate and maintain Reed Sternberg cells have been previously identified, these cells have the stem cell marker ALDH and are CD27 + ve but share the clonal immunoglobulin gene recombination with the Reed Sternberg cells [61] confirming the lack of a hierarchical structure.

### Other mature B cell malignancies

We have previously hypothesised that the relationship to the onset of malignancy and the relationship with the VDJ or SHM activity determines much of the sensitivity of the malignant cells to chemotherapy and would argue that this degree of sensitivity is maintained in the stochastic cancer stem cells arising from these malignancies. The malignancies closely linked to VDJ and SHM, ALL, DLBCL, Hodgkin’s disease and Burkitt’s lymphoma are routinely curable with chemotherapy. However in contrast in malignancies arising distant from these genetic events; CLL, mantle cell lymphoma and multiple myeloma are not chemotherapy curable. We would argue that the apoptotic sensitivity linked to VDJ and SHM is absent in these cells distant from these events so determining the lack of extreme chemotherapy sensitivity in both the malignant cells and their counterpart stochastic stem cells.

### In gestational trophoblast tumours

The pregnancy associated malignancies of post molar pregnancy trophoblast tumour and gestational choriocarcinoma are rare but highly curable with chemotherapy treatment [62, 63]. The model of cancer stem cells is perhaps less relevant to these cells and their malignancies as they arise from the initial cells that are formed at fertilisation and nuclear fusion. Whilst in this situation there can be no hierarchical cancer stem cells, as these cells arise from the first point in embryological development, recent work has indicated the presence of cancer cells with stem like properties developing within trophoblast cell lines [64]. We would suggest that the cells with stem like properties in this malignancy will share the innate sensitivity to chemotherapy that the trophoblast cells have naturally due to their close temporal relationship with the unique genetic event of nuclear fusion and the associated apoptotic sensitivity [4].

### In germ cell tumours

Germ cell tumours are rare and predominantly arise from the pre-malignant precursors carcinoma in situ (CIS) in men and gonadoblastoma in women [65, 66]. Both of these contain fetal gonocytes that have matured incorrectly and have an altered balance of the meiotic/mitotic switch [67]. The malignant cells that arise from these cells are extremely sensitive to chemotherapy and patients with advanced germ cell tumours are routinely cured. Similarly to the other chemotherapy curable malignancies these cells are biologically isolated from the standard tissue specific stem cell, the spermatogonial stem cell as a result of their developmental pathway. Whilst testicular cancer cells with stem cell like properties have been described, they appear to be extremely sensitive to chemotherapy and radiation [68]. These cells also arise in a stochastic non-hierarchical way from the cancer cells themselves and we would hypothesis that the germ cell cancer stem cells share the cancer cells innate apoptotic sensitivity to chemotherapy resultant from their developmental proximity to the meiotic pathway [4].

### In the childhood malignancies

The childhood malignancies have less clear developmental pathways but are generally believed to arise from embryological cells with blocked and failed development. We have previously postulated that the high apoptotic potential and chemotherapy curability of these rare malignancies is linked to the development of the malignant phenotype and blocked development occurring soon after gastrulation [4].

In neuroblastoma the malignant cells are primitive cells with blocked embryological development that prevents normal cellular differentiation [69]. Cells with cancer stem properties have been identified within the tumour cells and these cells can self renew, differentiate to all of the constituent parts of neuroblastoma and effectively produce tumours in mice [70]. In Wilms’ tumours recent studies have indicated that the cancer stem cells are not early renal stem cells but are a more developed cell that can serve to de-differentiate to produce the full repertoire of cells seen in the malignancy [71]. Similarly in the other childhood chemotherapy curable malignancies, Ewing’s sarcoma and osteosarcoma the cancer stem cells that have been identified do not follow the conventional hierarchical model with Ewing’s potentially arising from a derivative of a mesenchymal stem cell [72].

The finding that these other potentially chemotherapy curable malignancies do not arise in the conventional hierarchical manner from cancer stem cells related to standard tissue specific stem cells, also helps explain why these tumours frequently do not relapse after successful chemotherapy treatment.

## Each of the chemotherapy curable malignancies lacks hierarchical cancer stem cells

In the chemotherapy curable malignancies, the cells of each diagnosis arise from a cell which would normally have a transient passage through a complex developmental stage during which they become malignant. In health, a normal cell does not persist long term as a new trophoblast cell, or as a pro-B cell or germinal centre B cell, similarly the process of meiosis occupies only a relatively brief step in the complete pathway of production of sperm from sperm stem cells. In health cells passing through these developmental stages, do so for a relatively short length of time, and then exit to either the next stage in their development after their unique genetic event successfully occurs or naturally undergo apoptosis in response to event related DNA damage or an absence of positive selection.

In the common solid malignancies the hierarchical relationship of the tissue specific somatic stem cell, the cancer stem cell and the cancer cells is relatively simple with the tissue specific stem cell giving rise through mutation to the cancer stem cell and the cancer stem cells in turn giving rise to the more rapidly growing standard cancer cells [73].

In contrast in the chemotherapy curable malignancies the relationship and role of cancer stem cells is more complex and at present less clearly defined. The biological difference is most easily seen in the B cell and T cell malignancies where there is a very differing relationship with their stem cells than most conventional malignant cells. Here the stem cell that is the original source of cells for all lymphoid and myeloid malignancies is the haematopoietic stem cell (HSC). However this cell, which is very resistant to chemotherapy, only acts in the conventional stem situation for the initial lead off into B cell, T cell and myeloid cell development. After this point the B cell and T cells undergo the complex genetic recombinations of V(D)J genes and for the B cells somatic hypermutation and class switching that lead to their specific clonal identity. These processes in which DNA is cut, rearranged, mutated, rejoined and repaired contain much of the risk of DNA damage that leads to leukamagenesis and lymphomagenesis [74] but also provide the pathways that allow these malignancies to be chemotherapy curable [3].

The malignancies that arise further along these pathways and more distant from the HSC are physiologically very different cells and now clonally and mutationally only very distantly related to the original HSC. Whilst HSC that carry mutations have been recognised to confirm a risk of development of CLL, these cells are not directly clonally related to a current diagnosis of CLL and cannot serve to repopulate the pool of established CLL cells after chemotherapy treatment [75]. Whilst it is apparent that B cell and T cell malignancies cannot have cancer stem cells in the same hierarchical way that the common cancers may do, it is postulated that leukemia, lymphoma and other lymphoid malignancies can contain cells with stem like properties that are derived in a stochastic method from the malignant cells that arise at the key differing points along the B cell and T cell development pathway [58].

In a similar fashion there is a similar lack of conventional hierarchical cancer stem cells for the other chemotherapy curable malignancies. In the gestational tumours the malignant cells arise from the primitive trophoblast cells, which are present for only short time after conception and nuclear fusion. Similarly the malignant germ cell tumours have no healthy counterpart and are believed to arise from arrested gonocytes that having ongoing meiosis/mitosis stresses. These cells are not present in health and the malignant germ cell tumour cells are not supported by stem cells arising from the spematogonial stem cells.

These biological observations suggests that the chemotherapy curable malignancies share a common theme in that they do not have conventional hierarchical cancer stem cells. In each case their cancer stem cells appear to arise either from within the cancer cell pool in the stochastic model, as in ALL, lymphoma and choriocarcinoma, or from a persistent embryonal cell with blocked development as in the childhood malignancies and germ cell tumours. In Table 1 a summary of chemotherapy curability of the key tumour types along with their hierarchical stem cell status and relationship to the malignancy associated genetic events is demonstrated.

### Chemotherapy curable and non-curable B cell malignancies and stochastic cancer stem cells

This observational model on the relationship between the chemotherapy curability of cancer cells and their stem cells is complicated in that some types of B cell and T cell malignancies including CLL, follicular NHL, multiple myeloma and the mature T cell malignancies also will share the same non-hierarchical stem cell model, frequently respond well to chemotherapy treatment but are not curable with chemotherapy.

**Fig. 2:**
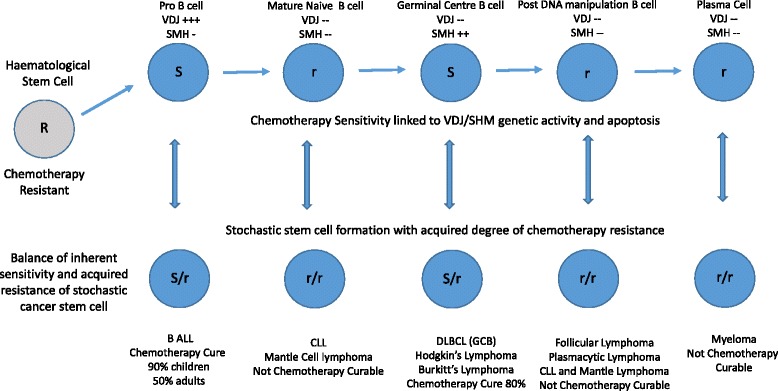
The Properties of Stochastic Stem Cells in B Cell Malignancies. In B cell malignancies the main cancer mutational events occur during VDJ recombination and somatic hypermutation. The resultant malignant cells that arise during B cell development remain fixed at that point in development and neither progress to be terminally differentiated plasma cell or undergo apoptotic death as B cells that fail developmental checks normally do. Dependant on the timing of the onset of the malignant phenotype the B cell takes on the phenotype of the cell of origin. Each of the malignant cell types are developmentally isolated from the HSC but can develop stochastic cells with stem like qualities, including a degree of resistance to chemotherapy. We suggest that there is a balance of this stem like acquired chemotherapy resistance against the apoptotic sensitivity linked to the upregulated activity in B cell development related to VDJ and SHM activity. This balance determines if the differing stochastic stem cells will survive chemotherapy and hence if the B cell malignancy can have the potential to be cured with chemotherapy or not

In this situation we would argue that the characteristics of the stochastic stem cells with regard to DNA damage and induction of apoptosis is not determined solely by the process of their development of stem cell characteristics but is also very significantly affected by the intrinsic characteristics of the malignant cell from which they arise [53]. Figure 2 shows the potential impact of this in the differing chemotherapy curability of the B cell malignancies. The stochastic stem cells arising from ALL will still have the apoptotic sensitivity linked to VDJ activated, similarly the stochastic stem cells in DLBCL will still have the SHM associated apoptotic sensitivity. In contrast in the stochastic stem cells arising from CLL, follicular lymphoma and multiple myeloma cells in which these processes are not active, will not have this overpowering apoptotic sensitivity and are able to resist chemotherapy induced damage and can serve to repopulate the malignancy after chemotherapy treatment. We would hypothesise that the ongoing activity of these genetic recombination related apoptotic pathways are sufficiently active to overcome the degree of resistance associated with development of stemness in the stochastic cancer stem cell and leave these biologically unique cancer stem cells sensitive to chemotherapy treatment.

## Conclusions

The ability of simple cytotoxic DNA damaging chemotherapy drugs used as single agents or more commonly in combination to cure a select group of relatively rare malignancies has been one of the great achievements from the early days of cancer therapeutics in the 1950–70s. However since then, despite enormous endeavour, no additional metastatic malignancy has been added to the short list of routinely curable metastatic cancers.

This dramatic divergence in responsiveness between different malignancies to chemotherapy treatment and how to improve it, has been one of the major areas of oncology research. In this paper we have reviewed how the cancer stem cell structure associated with these malignancies may impact on this divergence.

Our observations indicate that each of the chemotherapy curable malignancies arise in a cell type that has a transient passage through a complex developmental stage involving complex DNA manipulation. The conventional hierarchical cancer stem cell system is absent in each of the chemotherapy curable malignancies and these factors are likely to be an important associations with the dramatic and divergent responses seen to chemotherapy. Additionally each of the chemotherapy curable cancers arises from a parent cell that has much lower levels of ABC transporter protein expression than hierarchical cancer stem cells have is also likely to lead to a further increase in efficacy of chemotherapy treatment.

In the chemotherapy curable malignancies the cancer stem cells where present, generally arise in a non-hierarchical or stochastic method from within the pool of tumour cells itself. In each malignancy these cells are either developmentally isolated from their originating tissue specific somatic stem cells or have no conventional hierarchical cancer stem cells. As a result of the absence of a hierarchical cancer stem cells, the chemotherapy curable malignancies can only have cells with stem cell like properties that have arisen from with the cancer cell pool. These cells will take much of their properties from the cancer cells rather than having the propertices of a conventional cancer stem cell that is closely related to the damage and chemotherapy resistant somatic stem cells.

We have previously postulated the potential importance regarding the presence of the key cellular pro-apoptotic sensitivity associated with the unique genetic events of nuclear fusion, VDJ recombination, somatic hypermutation, meiosis and gastrulation. Normal healthy cells undergoing these activities are exceptionally sensitive to the action of chemotherapy drugs, as seen in the action of methotrexate in producing pregnancy loss [76], of chemotherapy in removing CIS [77] and the enormous sensitivity of healthy early B cells to cytotoxics [54, 78]. The ongoing activity of the apoptotic pathways associated with these processes in cells frozen at these points in development could be an important determinant of chemotherapy curability in the malignancies arising in them [3, 4].

We would argue that this association also directly impacts on the chemotherapy sensitivity of the non-hierarchical or stochastic cancer stem cells associated with these varied malignancies. In this situation the responsiveness to chemotherapy of the stochastic cancer stem cell will be determined by both the acquired characteristics of stemness but also significantly impacted by the underlying apoptotic sensitivity of the malignant cell. As a result, in the chemotherapy curable malignancies the degree of chemotherapy resistance that the adoption of stemness gives to these stochastic cancer stem cells, is functionally outweighed by the apoptotic sensitivity and allows the chemotherapy to kill the stem cells. In contrast in the similar malignancies of CLL/mantle cell lymphoma/myeloma/low grade NHL which also have stochastic stem cells and no hierarchical stem cells, their stochastic stem cells do not have this extreme apoptotic sensitivity and their stem cells are able to survive the chemotherapy, in a similar fashion to standard hierarchical cancer stem cells.

In this debate section, it is clear that there is a great deal of data needed to confirm or repudiate this new hypothesis. To date there has been only limited research on these natural apoptotic pathways, their mechanisms and the controls occurring in these cells. Additionally there has been also limited work on the differential apoptotic responses of the chemotherapy curable malignancies compared with the incurable. However whilst this paper may serve as a prompt to explore these areas in more depth it should also serve to remind that the genes that can give rise to overwhelming apoptosis in response to chemotherapy are present in every cancer cell and in their respective cancer stem cell. The key of how to unlock this potential is awaited.

## Abbreviations

ABC: ATP-binding cassette
ALDH: Alcohol dehydrogenase
ALK: Anaplastic lymphoma kinase
ALL: Acute lymphoblastic leukemia
AML: Acute myeloid leukaemia
CIS: Carcinoma in situ
CLL: Chronic lymphocytic leukemia
DNA: Deoxyribonucleic acid
HSC: Hematopoietic stem cells
SHM: Somatic hypermutation
VDJ: Variable/diversity/joining
